# Constipation and high blood pressure variability

**DOI:** 10.1038/s41440-023-01514-5

**Published:** 2023-11-20

**Authors:** Eikan Mishima

**Affiliations:** 1 Division of Nephrology, Rheumatology and Endocrinology, Tohoku University Graduate School of Medicine, Sendai, Japan; 2 Institute of Metabolism and Cell Death, Helmholtz Zentrum München, Neuherberg, Germany

**Keywords:** Hypertension, Bowel movement, Cardiovascular events, Autonomic nerve system, Microbiota

Blood pressure variability has recently gained increased attention as an independent risk factor for adverse cardiovascular events and hypertensive target organ damage, including chronic kidney disease and stroke [1, 2]. Consequently, achieving the stabilization of blood pressure variability, encompassing day-to-day, diurnal, and seasonal fluctuations, is desired for effective blood pressure control to mitigate associated risks, in addition to managing high blood pressure itself [3, 4].

Blood pressure variability is mainly regulated by the function of autonomic nerve systems. Given that autonomic function governs heart rate and vascular constriction, dysregulation of autonomic function is linked to elevated blood pressure variability [5]. For instance, autonomic dysfunction disorders, such as diabetic neuropathy and Parkinson’s disease, often present with substantial blood pressure variability. Moreover, since autonomic function influences bowel movements, constipation is a common symptom of such diseases. Thus, both blood pressure variability and defecation status serve as indicators of autonomic function, potentially influencing each other or occur in parallel. Notably, clinical studies have reported a correlation between poor defecation habits, including constipation, and an increased risk of cardiovascular events and chronic kidney disease [6–9]. Conversely, patients with hypertension have shown a higher prevalence of chronic constipation [8]. Additionally, the relationship between hypertension and the intestines involves the gut microbiota, which plays a key role in influencing the pathophysiology of hypertension, the therapeutic effects of antihypertensive agents, and the regulation of the sympathetic nervous system [10].

**Fig. 1:**
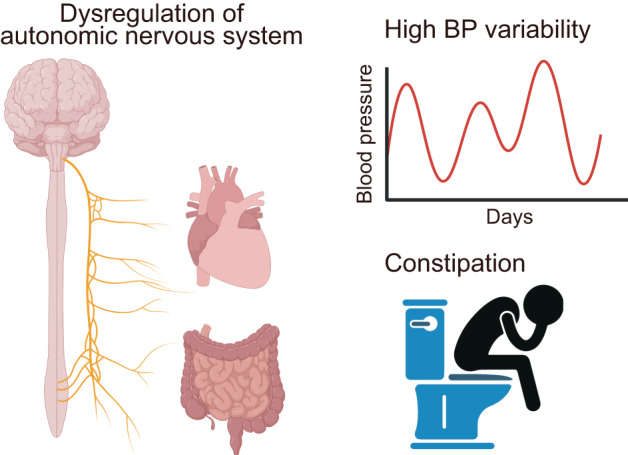
Defecation status and blood pressure variability. The autonomic nervous system plays an important role in regulating both blood pressure (BP) variability and bowel movement. The association of elevated BP variability and constipation, shown in this study, may be indicative of autonomic dysfunction involved in gut-vascular axis

In the recent issue of *Hypertension Research*, Kubozono et al. conducted an observational study examining the association between day-to-day blood pressure variability and defecation status using data from a community cohort of the Japanese general population [11]. In this study, home blood pressure data from a total of 184 subjects, with an average age of 71 years, were analyzed at baseline and one year later, and the day-to-day variability of systolic blood pressure was assessed. The results demonstrated that constipation, defined as a defecation status of less than once per day, was independently associated with an elevated coefficient of variation in day-to-day blood pressure, indicating increased blood pressure variability, although no significant differences were observed in the mean home blood pressure values between participants with and without defecation issues. Furthermore, the proportion of participants with elevated day-by-day blood pressure variability at one year was significantly higher in the constipation group compared to the non-constipation group. Based on these findings, the authors concluded that constipation is independently associated with elevated day-to-day blood pressure variability (Fig. 1).

In considering the influence on blood pressure variability and defecation status, it is essential to note that several classes of antihypertensive agents, such as calcium channel blockers, β-adrenoceptor antagonists, and diuretics, can lead to constipation as an adverse effect [12]. Moreover, antihypertensive medication can impact the degree of blood pressure variability. Importantly, poor adherence to prescribed medication considerably contributes to increased day-to-day blood pressure variability. However, in the present study, only 10% of the subjects were prescribed antihypertensive agents [11]. Furthermore, among the group of individuals not taking antihypertensive medications, no daily bowel movement status demonstrated an association with blood pressure variability. Thus, in the present data set, clinical characteristics of the subjects may play a more important role in blood pressure variability than the use of antihypertensive drugs. These findings underscore the intriguing relationship between constipation and elevated blood pressure variability, presumably linked to dysregulated autonomous nerve functions.

Since this study is observational, a causal relationship between constipation and elevated blood pressure variability cannot be established. Nevertheless, if infrequent bowel movements can be improved, it may be possible that blood pressure variability might also improve. Consequently, therapeutic interventions aimed at improving defecation status through the use of laxatives or lifestyle interventions may hold the potential to stabilize blood pressure variability, potentially reducing the future risk of cardiovascular events and hypertensive organ damage. Further studies are warranted to determine whether interventions, including medication and lifestyle modifications against constipation, can ameliorate the intestinal environment and reduce blood pressure variability via gut-vascular axis.
